# Neuroprotective effect of Dl-3-n-butylphthalide against ischemia–reperfusion injury is mediated by ferroptosis regulation *via* the SLC7A11/GSH/GPX4 pathway and the attenuation of blood–brain barrier disruption

**DOI:** 10.3389/fnagi.2023.1028178

**Published:** 2023-02-23

**Authors:** Shuangli Xu, Xuewei Li, Yutian Li, Xiangling Li, E. Lv, Xiaojun Zhang, Youkui Shi, Yanqiang Wang

**Affiliations:** ^1^Emergency Department, The Affiliated Hospital of Weifang Medical University, Weifang, Shandong, China; ^2^Department of Rheumatology, The Affiliated Hospital of Weifang Medical University, Weifang, Shandong, China; ^3^School of Pharmacy, Weifang Medical University, Weifang, Shandong, China; ^4^Department of Internal Medicine, The Affiliated Hospital of Weifang Medical University, Weifang, Shandong, China; ^5^Department of Histology and Embryology, Weifang Medical University, Weifang, Shandong, China; ^6^Department II of Neurology, The Affiliated Hospital of Weifang Medical University, Weifang, Shandong, China

**Keywords:** N-butylphthalide, ischemic/reperfusion, ferroptosis, blood–brain barrier, neuroinflammation

## Abstract

**Background:**

Stroke is one of the most severe diseases worldwide, resulting in physical and mental problems. Dl-3-n-butylphthalide, a compound derived from celery seed, has been approved for treating ischemic stroke in China. No study has evaluated how Dl-3-n-butylphthalide affects the ferroptosis SLC7A11/GSH/GPX4 signal pathway and blood–brain barrier (BBB) PDGFRβ/PI3K/Akt signal pathways in the rat middle cerebral artery occlusion/reperfusion (MCAO/R) model of ischemic stroke.

**Methods:**

Sprague–Dawley rats were used to develop the MCAO/R model. Our study used three incremental doses (10, 20, and 30) of Dl-3-n-butylphthalide injected intraperitoneally 24 h after MCAO/R surgery. The neuroprotective effect and success of the model were evaluated using the neurofunction score, brain water content determination, and triphenyl-tetrazolium chloride-determined infarction area changes. Pathological changes in the brain tissue and the degree of apoptosis were examined by hematoxylin and eosin, Nissl, and terminal deoxynucleotidyl transferase dUTP nick end labeling staining. In addition, pathway proteins and RNA expression levels were studied to verify the effects of Dl-3-n-butyphthalide on both pathways. At the same time, commercial kits were used to detect glutathione, reactive oxygen species, and malondialdehyde, to detect oxidative stress in brain tissues.

**Results:**

The middle dose of Dl-3-n-butylphthalide not only improved MCAO-induced brain dysfunction and alleviated pathological damage, brain inflammatory response, oxidative stress, and apoptosis but also protected against ferroptosis and reduced BBB damage. These changes resulted in improved neurological function in the cerebral cortex.

**Conclusion:**

We speculate that Dl-3-n-butylphthalide has a neuroprotective effect on focal cerebral ischemia/reperfusion, which may be mediated through ferroptosis-dependent SLC7A11/GSH/GPX4 signal pathway and PDGFRβ/PI3/Akt signal pathway.

## 1. Introduction

Stroke is an acute cerebrovascular disease characterized by high mortality and morbidity and can cause focal neurological dysfunction. Ischemic stroke comprises about 87% of all stroke cases and is characterized by blocked blood vessels resulting in insufficient oxygen and nutrient delivery to brain tissue within minutes of the attack (Yan W. et al., 2021). Cerebral ischemia can cause a series of complex enzyme cascades, including inflammatory responses, energy metabolism disorders, oxidative stress, and disruption of the blood–brain barrier (BBB; Zhu et al., 2021). The primary clinical treatment for ischemic stroke involves thrombolytic drugs, antiplatelet aggregation drugs, and anticoagulants. However, thrombolytic therapy must be carried out within a limited treatment window, dramatically limiting its clinical application (Derek Barthels, 2020; Wei et al., 2021).

Dl-3-n-butylphthalide (DL-NBP) is a relatively new drug independently developed in China to treat cerebrovascular diseases. Its efficacy and safety have been evaluated in several clinical trials in China (Fan et al., 2021; Tan et al., 2021; Zhu et al., 2021).

Ferroptosis is a form of cell death that is distinguishable from apoptosis and autophagy (Yan H. F. et al., 2021). Since its discovery in 2012, it has become a research hotspot in the pathogenesis of various diseases. It is characterized by the production of reactive oxygen species (ROS) and lipid peroxidation (Hirschhorn and Stockwell, 2019). Cystine is transported by cysteine glutamate antiporter (system xc-) which consists of SLC7A11 in cells for the synthesis of glutathione (GSH) (Koppula et al., 2021). GSH is an important intracellular antioxidant and a substrate for the synthesis of GSH peroxidase 4 (GPX4). GPX4 converts lipid peroxides to non-toxic alcohols to protect cellular lipid peroxidation and is a key regulator of ferroptosis (Chen et al., 2021). SLC7A11, GSH, and GPX4 constitute the important ferroptosis signal pathway. Whether DL-NBP plays a role in ferroptosis and its main signaling pathway SLC7A11/GSH/GPX4 (solute carrier family 7 member 11/glutathione/glutathione peroxidase 4) in ischemic brain injury has not been reported.

Stroke caused damage to the BBB by a complex mechanism of injury and is regulated by a variety of mechanisms (Derek Barthels, 2020). Platelet-derived growth factor-BB (PDGF-BB), one of the therapeutic targets for endothelial dysfunction of the BBB, can bind to the PDGFβ receptor (PDGFRβ) and activate phosphatidylinositol-4,5-diphosphate 3-kinase/protein kinase B (PI3K/Akt), which is involved in neuroprotection against cerebral ischemic injury (Zheng et al., 2019). However, whether DL-NBP influences the PDGFR-β/PI3K/Akt signaling pathway is unknown and requires elucidation.

In this study, we evaluated the neuroprotective effect of DL-NBP in a model of ischemic reperfusion injury, and established and investigated its underlying mechanisms by analyzing the expression of important genes and proteins in the SLC7A11/GSH/GPX4 and PDGFR-β/PI3K/Akt signaling pathways.

## 2. Materials and methods

### 2.1. Animals

8-week-old Sprague–Dawley (SD) rats (weight 250–280 g) were bought from Jinan Peng Yue Experimental Animal Reproduction Company, Ltd. (Jinan, China) and were fed with pure water and sterile fodder in the Clinical Medicine Research Center of the Affiliated Hospital of Weifang Medical University. The rats’ cages were temperature (20°C–25°C) and humidity (60%–70%) controlled under a 12 h/12 h light/dark cycle. Our experimentations on animals were authorized by the Institutional Animal Care and Use Committee of Weifang Medical University and every attempt was made to reduce damage to the animals during the experiments. The animal study protocol was reviewed and approved by the experimental animal Ethics Committee of Weifang Medical University (2018–037).

### 2.2. Animal models

The middle cerebral artery occlusion/reperfusion (MCAO/R) rat model was used to carry out the research (Fluri et al., 2015). Rats were anesthetized with pentobarbital sodium (40 mg/kg) by intraperitoneal injection and the pain reflex was detected by the Randall-Selitto deep pressure test (calipers applied to the hind paw of the rat) in the perioperative period. Surgery was performed after the pain reflex disappeared. Briefly, the rat underwent a neck incision, exposing the right external carotid artery (ECA), internal carotid artery (ICA), and common carotid artery (CCA). After ligation of the distal ECA and proximal CCA, we clipped the ICA and made a small cut at the distal end of the CCA ligation. A thread was inserted (0.38–0.40 mm; MSRC40B200PK50, Shen Zhen RWD Life Science Co., Ltd., Shenzhen, China) with a thick head to approximately 16–20 mm and fixed. After 2 h, the thread tail was pulled out but the head was retained to restore blood circulation. The skin incision was then sutured. Sham rats underwent the same procedure but without occlusion. A successful model could be judged by Horner syndrome in the left eye when the rats awakened, bending of its right forelimb on lifting the tail, and the ability to move in a circle as they moved autonomously on the ground. Rats with massive bleeding, subarachnoid hemorrhage, and premature death/drop-out were excluded after cerebral ischemia–reperfusion injury (Feng C. et al., 2020). A total of 198 SD rats were operated on, in this experiment, of which 155 were finally included in the experiment and 16 were excluded due to unsuccessful molding; the mortality rate was 13.64%.

### 2.3. Grouping and drug treatment

The male SD rats were divided into the following five groups using a table of random numbers according to the principle of simple random allocation, and the original liquid of DL-NBP is provided by CSPC NBP Pharmaceutical Co., Ltd.: (i) a Sham group, in which rats underwent a sham operation (Sham); (ii) the MCAO/R group, in which rats underwent MCAO/R; (iii) MCAO/R + low-dose N-butylphthalide group (10 mg/kg; MCAO/R + NBP-L); (iv) MCAO/R + medium-dose N-butylphthalide group (20 mg/kg; MCAO/R + NBP-M); (v) MCAO/R + high-dose N-butylphthalide group (30 mg/kg; MCAO/R + NBP-H). This allocation minimized any selection bias (Hirst et al., 2014; Schulz et al., 2018). We selected the appropriate dose of DL-NBP using body surface area conversion according to the drug instructions and clinical dosage. 24 h after the rats underwent the MCAO/R operation, the treatment was administered at each corresponding dose in each group through intravenous injection into the femoral vein. After 24 h of DL-NBP (CSPC NBP Pharmaceutical Co., Ltd., Shijiazhuang, China) administration, the rats were euthanized after behavioral tests, and the brain tissue was obtained for analysis.

### 2.4. Modified neurological severity scores

The 18-point Garcia grading score was used to measure neurological function in each group of rats (*n* = 6) to assess the behavior 24 h after the drug treatment (Sha et al., 2019). The mNSS test is scored out of 18 points and includes three movement tests and two sensory experiments. The results are interpreted as follows: 1–6, mild injury; 7–12, moderate injury; and 13–18, severe injury. The evaluators were blinded to the different group allocations of the rats.

### 2.5. Cerebral blood flow measurement

Under the premise of double-blind, the success of MCAO in rats was monitored by laser speckle flow imaging (Heeman et al., 2019). Briefly, after anesthesia and sterilization, and the skull is exposed. The skull is slowly polished by a high-speed electric skull drill until the epidural forms a 10x6mm skull window. Record the blood flow before and after MCAO using laser speckle flow imaging (SIM BFI-HR PRO, Wuhan, China).

### 2.6. Evaluation of cerebral edema

Brain water content was measured by the standard wet-dry method (Cao et al., 2016). The right cerebral hemisphere in each group (*n* = 6) was separated 24 h after the drug treatment and weighed to obtain the wet weight. The dry weight was then obtained by dehydrating the brain in an oven at 105°C for 72 h. The evaluators were blinded to group allocation. The brain water content was calculated as follows: (wet weight–dry weight) /wet weight.

### 2.7. 2,3,5-Triphenyltetrazolium chloride staining

The brain tissue from five groups (*n* = 3) was sliced into 2-mm thick coronal sections (a total of 6 slices) after freezing in a-20°C refrigerator for 20 min. The slices were placed in a pre-prepared 1% TTC (Sigma-Aldrich, St. Louis, MO, USA) solution. After 15–30 min, the slices were stained according to the presence of specific non-ischemic areas (light red) and ischemic necrotic tissue (white) (Sha et al., 2019). This process was done by a member blind to the grouping. Image J software was used to analyze the cerebral infarct volume.

### 2.8. Hematoxylin and eosin, Nissl, and terminal deoxynucleotidyl transferase dUTP nick-end labeling staining

After anesthetization, the rats, selected by single blinding from each group (*n* = 3) were perfused through the left cardiac apex with normal saline followed by 4% paraformaldehyde (PFA). The rats were decapitated post-perfusion and the brains were separated. The tissue was immersed in 4% PFA overnight at 4°C. The tissues were embedded in paraffin and cut into 5-μM thick sections. After dewaxing using xylene and hydration using gradient ethanol, along with ddH_2_O washing, we stained the specimen using HE, Nissl, and TUNEL (Sha et al., 2019). Finally, the sections were viewed and imaged using a fluorescence microscope (Leica, Wetzlar, Germany).

### 2.9. Evaluation of BBB permeability

Evans blue (EB) was used to evaluate BBB integrity at 24 h after the drug treatment. The rats were picked by single blinding from each group. EB dye (2% in saline, 4 ml/kg, Solarbio, Beijing, China, n = 4) was injected through the right femoral vein 2 h before the brain was collected (Wang et al., 2020). Blood and intravascular dyes were removed by perfusing saline *via* the left ventricle in the rats. The right infarction cerebral hemispheres were separated on ice and then homogenized for 24 h at a temperature of 60°C in 2 mL dimethylformamide. After centrifugation, the absorbance value of the supernatant was measured at 632 nm. The EB content was calculated by its standard curve. The frozen slice of the cerebrum tissue dyed by EB could be observed with blue excitation light (620 nm) under a fluorescence microscope (Zeiss, Oberkochen, Germany).

### 2.10. Superoxide dismutase, GSH, and malondialdehyde content

After obtaining brain tissue from rats obtained by the blinded selection, the brain cortex was isolated on ice to prepare brain tissue homogenate (*n* = 3). According to the manufacturer’s instructions, the concentrations of ROS (Affandi, Shanghai, China), MDA (Nanjing Jiancheng, Jiangsu, China), and GSH (Meimian, Chengdu, China) in the brain tissue homogenate were measured with commercial kits.

### 2.11. Western blot analysis

The smashed fresh brain tissues were selected blindly and RIPA Lysis Buffer (Beyotime, Haimen, China) and protease phosphatase inhibitors (PMSF, Beyotime) were mixed to fully ground. The protein concentration was measured using a bicinchoninic acid assay (Beyotime). The belt was transferred to the polyvinylidene fluoride membrane (Beyotime) after an electrophoresis process. Membranes were blocked with 5% skim milk blocking buffer at 37°C for 2 h and incubated with the following ferroptosis-rand BBB-related primary antibodies: anti-SLC7A11 (Abcam, Cambridge, UK; 1: 5,000), anti-GPX4 (Abcam; 1: 1,000), anti-PDGFR-β (Solarbio; 1: 1,000), anti-TFR1 (Abcam; 1: 5,000), anti-GSS (Abcam; 1: 5,000), anti-PI3K (Abcam; 1: 1,000), anti-p-PI3K (Abcam; 1: 1,000), anti-Akt (Abcam; 1: 10,000), anti-p-Akt (Abcam; 1: 1,000), and anti-β-actin (Abcam; 1: 5,000) as an internal control, at 4°C overnight in a thermostat shaker. After being washed by TBST (Tris-HCI buffer salt solution+Tween) buffer, all membranes were incubated with the second antibodies (Proteintech, Rosemont, IL, USA; 1: 5,000) at 37°C for 2 h. Immunoreactive membranes were processed with a chemiluminescence assay (Beyotime) (Wang et al., 2020). ImageJ software was used for analysis.

### 2.12. Immunofluorescence staining

The sections made from rats selected using single-blinding were incubated with anti-occludin (Solarbio) and anti-ZO-1 (Solarbio) antibodies at 4°C overnight (Jin et al., 2021). After being washed with PBS, they were incubated together with the fluorescence-conjugated secondary antibody (Solarbio) at 25°Cfor for 1 h. In addition, the vascular endothelial cell markers CD31 were co-immunostaining with Zo-1 and occludin to observe the BBB integrity. The histopathological changes in the brain could be observed using a fluorescence microscope. The results were analyzed using ImageJ software.

### 2.13. Quantitative real-time polymerase chain reaction assay

Total RNA was isolated from the brain tissues of rats selected by blind selection (n = 3) using an RNA extraction kit (Beyotime, Jiangsu, China). cDNA was generated by reverse transcription at 50°C for 15 min and 85°C for 5 min in T-RNA apparatus (Bio-rad, Hercules, CA, USA). The reaction system, including 10 μL 2xUltraSYBR Mixture, 0.4 μl of PCR Forward Primer (10 μM), 0.4 μL of PCR Reverse Primer (10 μM), 0.8 μL of cDNA template, and 8.4 μL of ddH_2_O was processed in PCR (Bio-rad) under the following conditions: 95°C for 10 min denaturation, followed by 40 cycles of 95°C for 15 s, and 60°C for 60 s (Liu et al., 2019). The primer sequences (Sangon Biotech, Shanghai, China) used for qRT-PCR are shown in Table 1.

**Table 1:** Primer sequences.

Primer sequences	
SLC7A11	Forward 5′-ATGCAGTGGCAGTGACCTTT-3′
	Reverse 5′-GGCAACAAAGATCGGAACTG-3′
GPX4	Forward 5′-TGTGTAAATGGGGACGATGCC-3′
	Reverse 5′-ACGCAGCCGTTCTTATCAATG-3′
TFRC	Forward 5′-AGTAGGAGCCCAGAGAGACGCTTGG-3′
	Reverse 5′-CACTCAGTGGCACCAACAGCTCCAT-3′
PDGFR-β	Forward 5′-GTGCTCACCATCATCTCCCT-3′
	Reverse 5′-ACTCAATCACCTTCCATCGG-3′

### 2.14. Statistical analysis

The quantitative data analysis is presented as the mean ± standard deviation (SD) in the presence of at least three independent experiments. T-tests were used for the comparison of the two groups. GraphPad Prism 9 was used to conduct all statistical analyses. *p* values <0.05 were considered statistically significant.

## 3. Results

### 3.1. Dl-NBP improves neurological scores and reduces brain water content as well as cerebral infarct volume in MCAO/R rat

**Figure 1:**
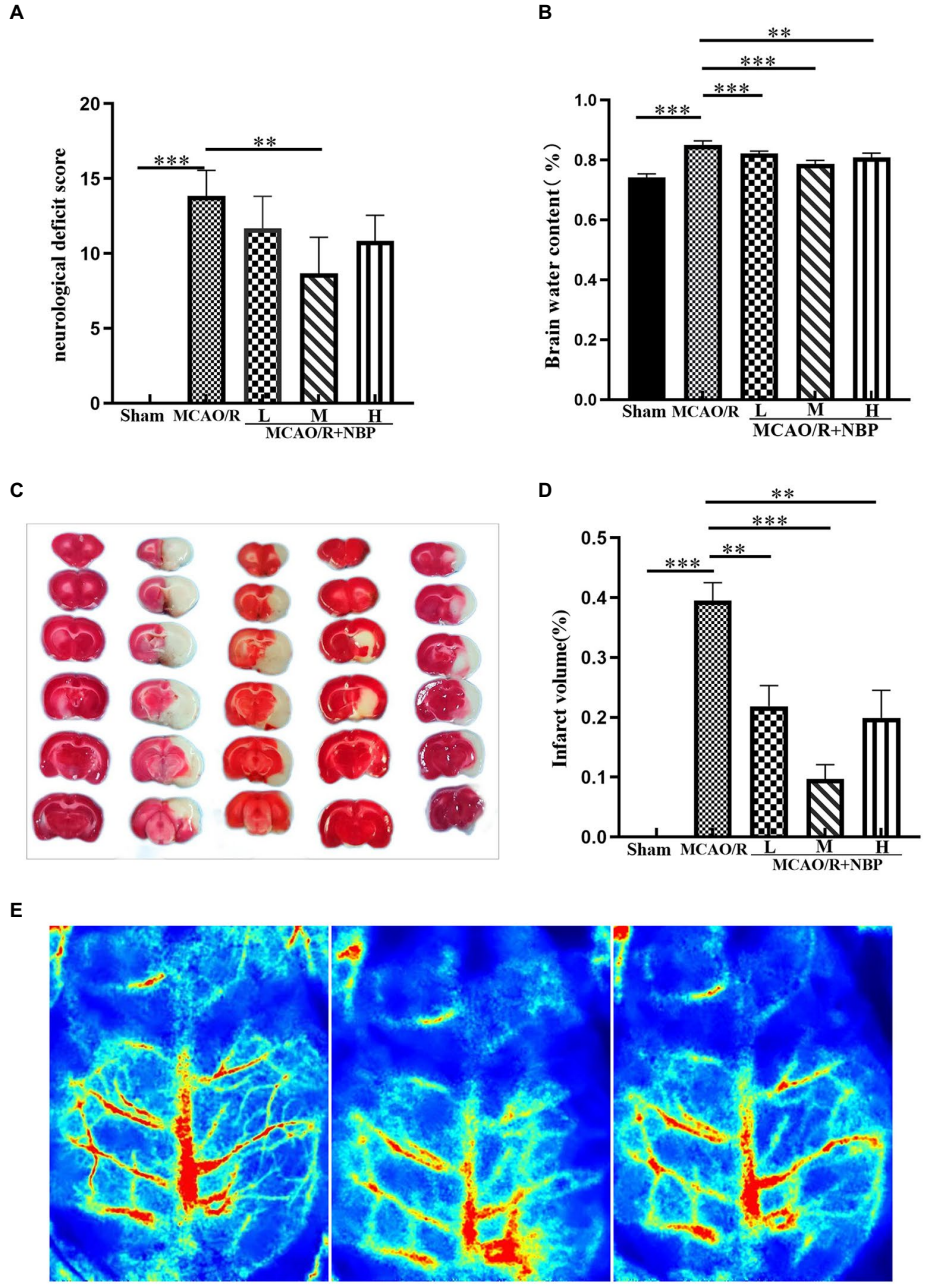
Effects of NBP on neurological deficit scores, brain water content, and cerebral infarct area. **(A)** Neurological deficit score. **(B)** Brain water content. **(C)** TTC staining. **(D)** Infarct volume. **(E)** Cerebral Blood Flow Measurement (From left to right, normal, ischemia and reperfusion). MCAO, middle cerebral artery occlusion; NBP, N-butylphthalide; TTC, 2,3,5-Triphenyltetrazolium chloride; MCAO/R + NBP-L, Low-dose N-butylphthalide, 10 mg/kg; MCAO/R + NBP-M, Medium-dose N-butylphthalide, 20 mg/kg; MCAO/R + NBP-H, High-dose N-butylphthalide, 30 mg/kg. *p < 0.05; **p < 0.01; ***p < 0.001. The values represent the mean ± SD, *n* = 6.

We observed the cerebral blood flow by laser speckle flow imaging system to determine the success of the MCAO/R rat model (Figure 1E). To examine whether NBP is helpful for neural function in rats with MCAO/R, a single-blind method was used to score each group of rats (n = 6) 24 h after the corresponding treatment. No significant changes were observed in the Sham group. Compared with the Sham group, rats in other groups showed significantly increased neurological deficit scores. The neurological deficit score in the MCAO/R + NBP-M group was significantly reduced compared to the Sham group (*p* < 0.01, 8.667 ± 2.422 vs. 13.83 ± 1.722; Figure 1A). After treatment with different doses of NBP in the three groups, the water content of the brain decreased significantly compared to the MCAO group (*p* < 0.01 or *p* < 0.001 vs. 0.8500 ± 0.01414; Figure 1B). Through TTC staining, rat cerebral infarction volume analysis showed that no injuries occurred in the Sham group, and white areas of infarction were observed in the remaining groups. Compared with the MCAO group, the cerebral infarct area in the NBP treatment group was significantly reduced (*p* < 0.01 or *p* < 0.001 vs. 0.3949 ± 0.03035; Figures 1C,D). The results showed that NBP effectively improved the neurological performance of MCAO model rats and reduced the cerebral infarction area and brain water content.

### 3.2. Dl-NBP protects against neuronal necrosis and apoptosis after MCAO/R in rat

**Figure 2:**
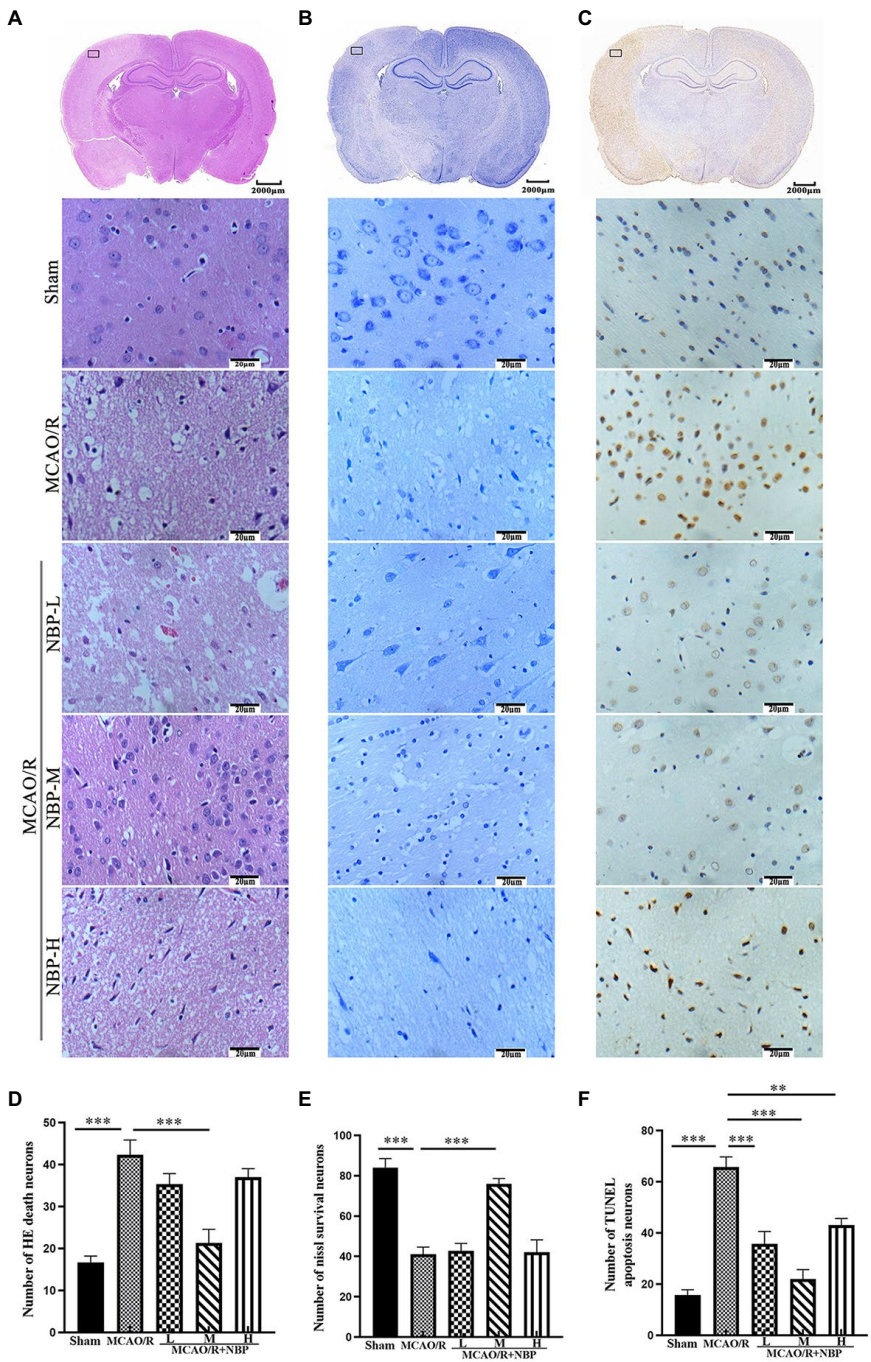
The histopathological and structural changes by HE, Nissl, and TUNEL staining. **(A)** HE staining. **(B)** Nissl staining. **(C)** TUNEL staining. **(D–F)** Quantitative analysis of the staining above (×400). HE, hematoxylin and Eosin; TUNEL, terminal deoxynucleotidyl transferase dUTP nick-end labeling. *p < 0.05; **p < 0.01; ***p < 0.001. Data are presented as the mean ± SD, *n* = 3.

HE staining of the coronal brain slices showed that the neuronal cells in the cerebral cortex in the Sham group were neatly arranged and structurally normal. In contrast, the damaged side of the brain in the remaining groups showed evident cell disorder, neuronal loss, a large amount of vacuole space, and partial nuclear dissolution and condensation (*p* < 0.001, Sham vs. MCAO; Figure 2A; Supplementary Figure 1A). Regarding quantitative analysis, neuronal pathology in the MCAO/R + NBP-M group improved significantly compared with the MCAO group (*p* < 0.001, 21.33 ± 3.215 vs. 42.33 ± 3.512; Figure 2D). Nissl bodies in the neurons were stained purple-blue in the cytoplasm and light blue in the nuclei (Song et al., 2022). Compared to the Sham group, the MCAO group had fewer Nissl bodies (*p* < 0.001, Sham vs. MCAO; Figure 2B; Supplementary Figure 1B). Quantitative analysis showed that the number of Nissl bodies in the medium DL-NBP treatment group increased compared with the MCAO group (*p* < 0.001, 76.00 ± 2.646 vs. 41.00 ± 3.606; Figure 2E). Apoptotic cells (brown-yellow staining) were observed in the cerebral cortex on the side of the infarction (Figure 2C). Less frequent neuronal apoptosis was observed in the DL-NBP compared to the MCAO groups (*p* < 0.001, 22.00 ± 3.606 vs. 65.67 ± 4.041; Figure 2F). These findings suggest that DL-NBP therapy improves MCAO-induced neuronal disorders, death, and apoptosis.

### 3.3. Dl-NBP reduces BBB damage after MCAO/R in rat

**Figure 3:**
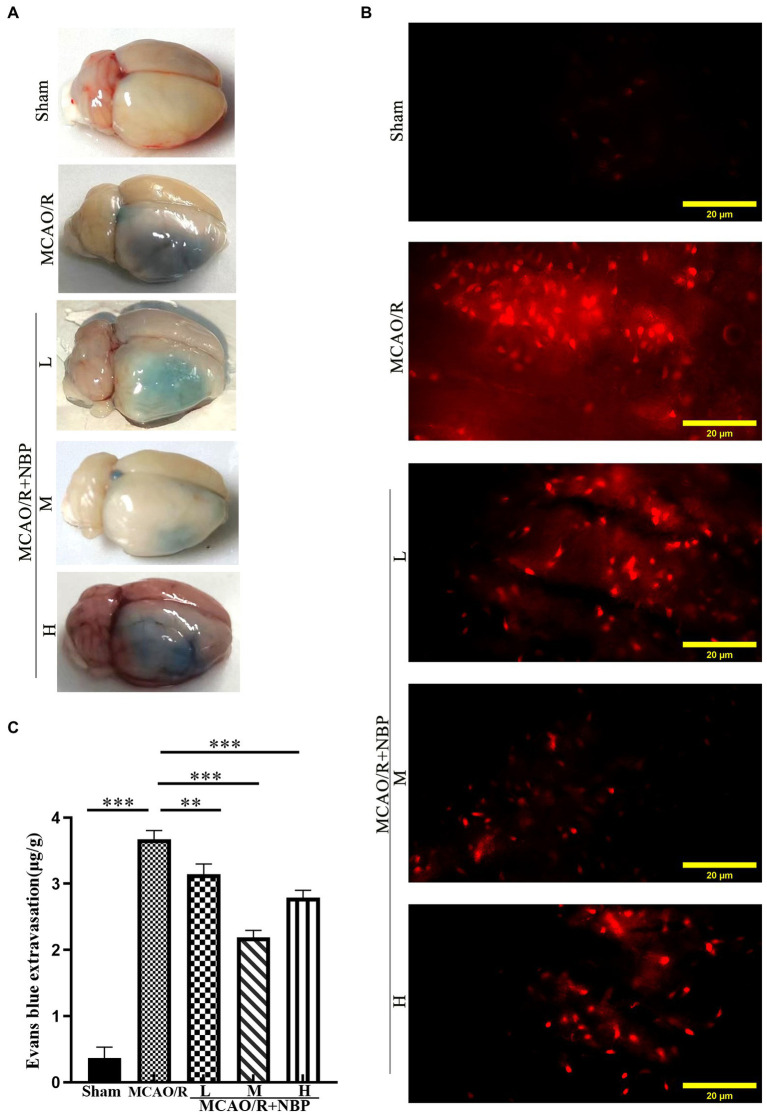
Evaluation of BBB Permeability. **(A)** The representative appearance of EB stained rats’ brains. **(B)** A fluorescence microscope observed the leakage of EB in rats’ frozen slices of brain tissue. **(C)** Quantitative analysis of EB content in brain tissue. EB, Evans blue; BBB, blood–brain barrier. *p < 0.05; **p < 0.01; ***p < 0.001. The values represent the mean ± SD, *n* = 4.

**Figure 4:**
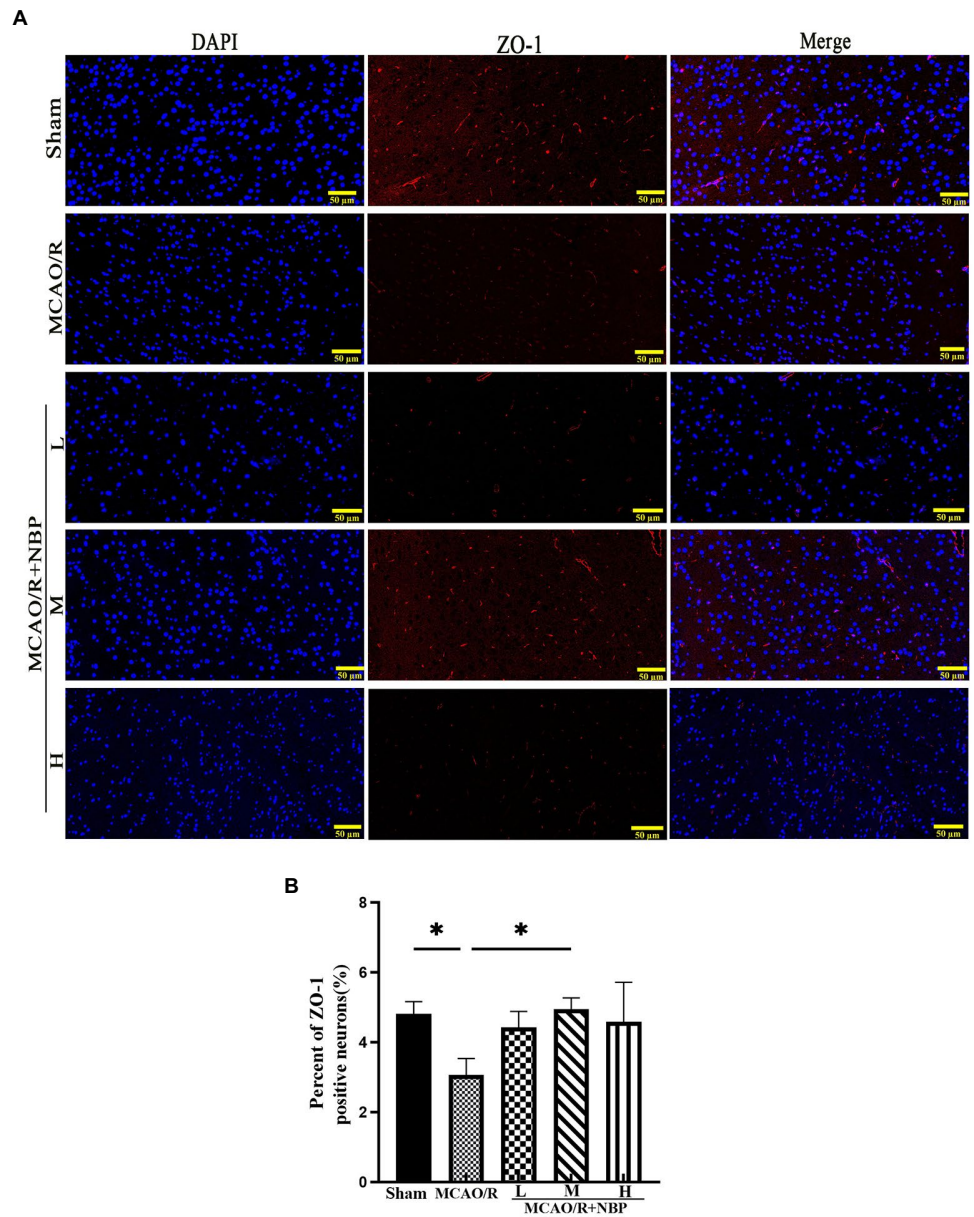
The ZO-1 tight junction protein of BBB. **(A)** The expression of ZO-1. **(B)** The qualification of ZO-1. BBB, the blood–brain barrier. *p < 0.05; **p < 0.01; ***p < 0.001. The values represent the mean ± SD, *n* = 3.

EB staining has been used to evaluate BBB permeability in ischemic hemispheres in the coronal plane (Guo et al., 2019) (Figure 3A). In the frozen slice of infarcted brain tissue, red dots appeared after excitation with a blue laser (excitation wavelength 620 nm) under a fluorescence microscope (Figure 3B). The MCAO group showed many red dots compared with the Sham group, while the red dots were significantly reduced after NBP treatment. EB content decreased after applying NBP (*p* < 0.001, 2.190 ± 0.1061 vs. 3.673 ± 0.1326; Figure 3C). To find out whether NBP treatment could affect BBB integrity, the tight junction proteins ZO-1 and occludin were observed by immunofluorescence staining (Figure 4A; Supplementary Figure 2A). In Supplementary Figures 3, 4, it is clear that the application of medium dose DL-NBP could protect BBB integrity compared to the MCAO/R group by labeling ZO-1 and occludin with vascular endothelial cell markers CD31 (Supplementary Figures 3–5). It was concluded that DL-NBP could protect the expression of ZO-1 (*p* < 0.05, 4.949 ± 0.3181 vs. 3.066 ± 0.4698; Figure 4B) and occludin (*p* < 0.001, 5.800 ± 0.4400 vs. 2.447 ± 0.2550; Supplementary Figure 2B).

### 3.4. The protective effect of DL-NBP on BBB may be mediated through PDGFRβ/PI3K/Akt signal pathway

**Figure 5:**
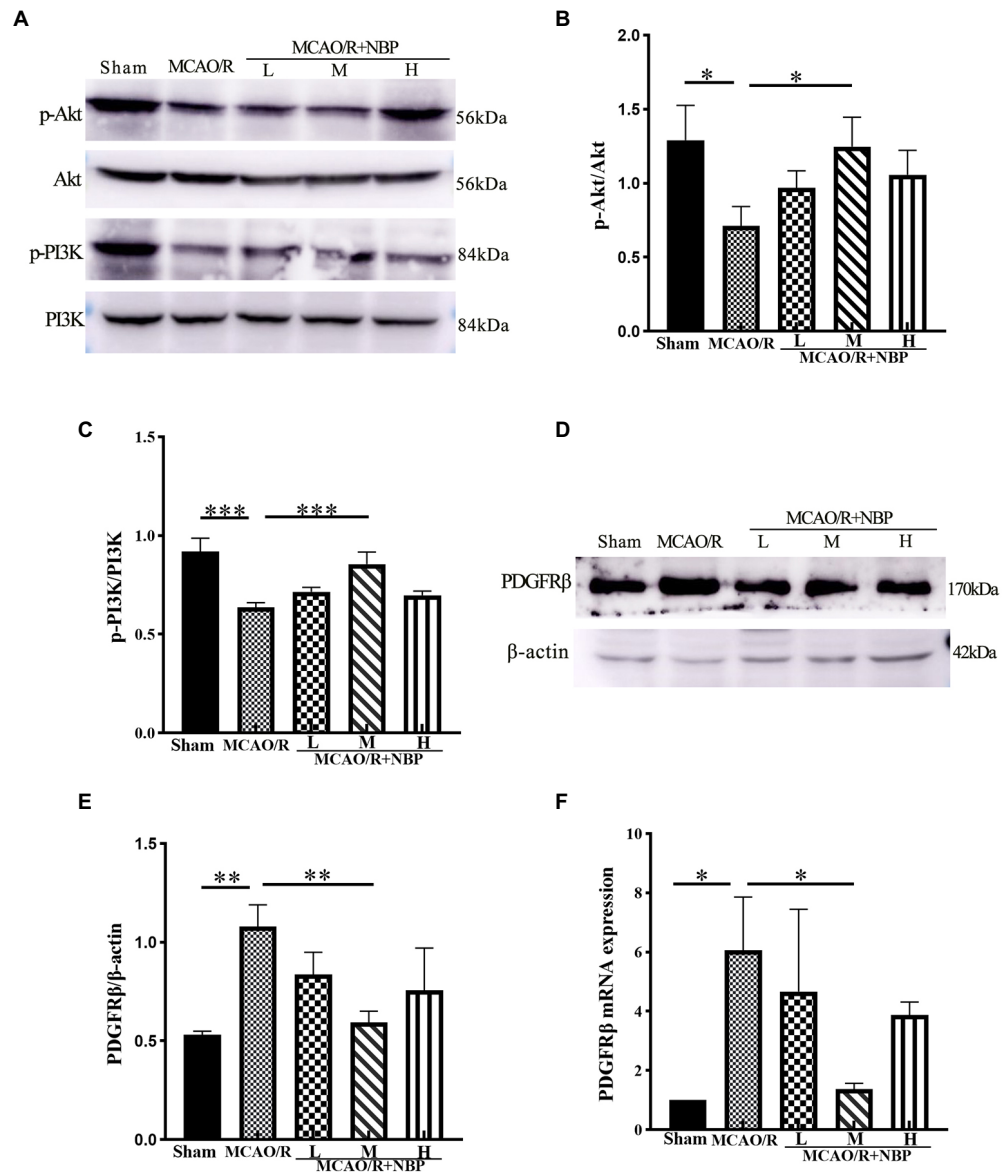
Western blot and PCR of BBB signal protein. **(A)** Representative western blot image of p-Akt, Akt, p-PI3K, and PI3k. **(B,C)** The expression ratio of p-Akt/Akt and p-PI3K/PI3k is quantified by image J software and is represented as a histogram. **(D)** Representative western blot image of PDGFRβ. **(E)** The expression ratio of PDGFRβ/β-actin is quantified by Image J software and is represented as a histogram. **(F)** PCR for PDGFRβ levels within cerebral cortex tissues. BBB, the blood–brain barrier; PCR, real-time polymerase chain reaction. *p < 0.05, **p < 0.01, ***p < 0.001. The values represent the mean ± SD, *n* = 3.

The expression of p-Akt and p-PI3K proteins in the MCAO group was significantly downregulated compared to that in the Sham group (*p* < 0.05, 0.7132 ± 0.1293 vs. 1.292 ± 0.2336, 0.6355 ± 0.02536 vs. 0.9193 ± 0.06814; Figures 5A–C). In addition, the expression of p-Akt and p-PI3K proteins in the MCAO/R + NBP-M group was significantly higher than in the MCAO group (*p* < 0.05, 1.247 ± 0.1993 vs. 0.7132 ± 0.1293, 0.8556 ± 0.0611 vs. 0.6355 ± 0.0254; Figures 5B,C). After MCAO, p-PDGFRβ expression was upregulated (*p* < 0.01, 1.079 ± 0.1097 vs. 0.5305 ± 0.01927; Figures 5D,E). Compared with the MCAO group, the PDGFRβ in the MCAO/R + NBP-M group was significantly lowered (*p* < 0.01, 0.5927 ± 0.05741 vs. 1.079 ± 0.1097; Figure 5E). Compared to the Sham group, MCAO group PDGFRβ (*p* < 0.05) levels rose in PCR. After applying DL-NBP, PDGFRβ (*p* < 0.05, MCAO/R + NBP-M vs. MCAO, 1.368 ± 0.1996 vs. 6.064 ± 1.800; Figure 5F) were reduced. These results are consistent with other experimental methods. These findings suggest that DL-NBP probably mediates the PDGFRβ/PI3K/Akt pathway to promote BBB integrity.

### 3.5. Dl-NBP protects against ferroptosis after MCAO/R in rat

**Figure 6:**
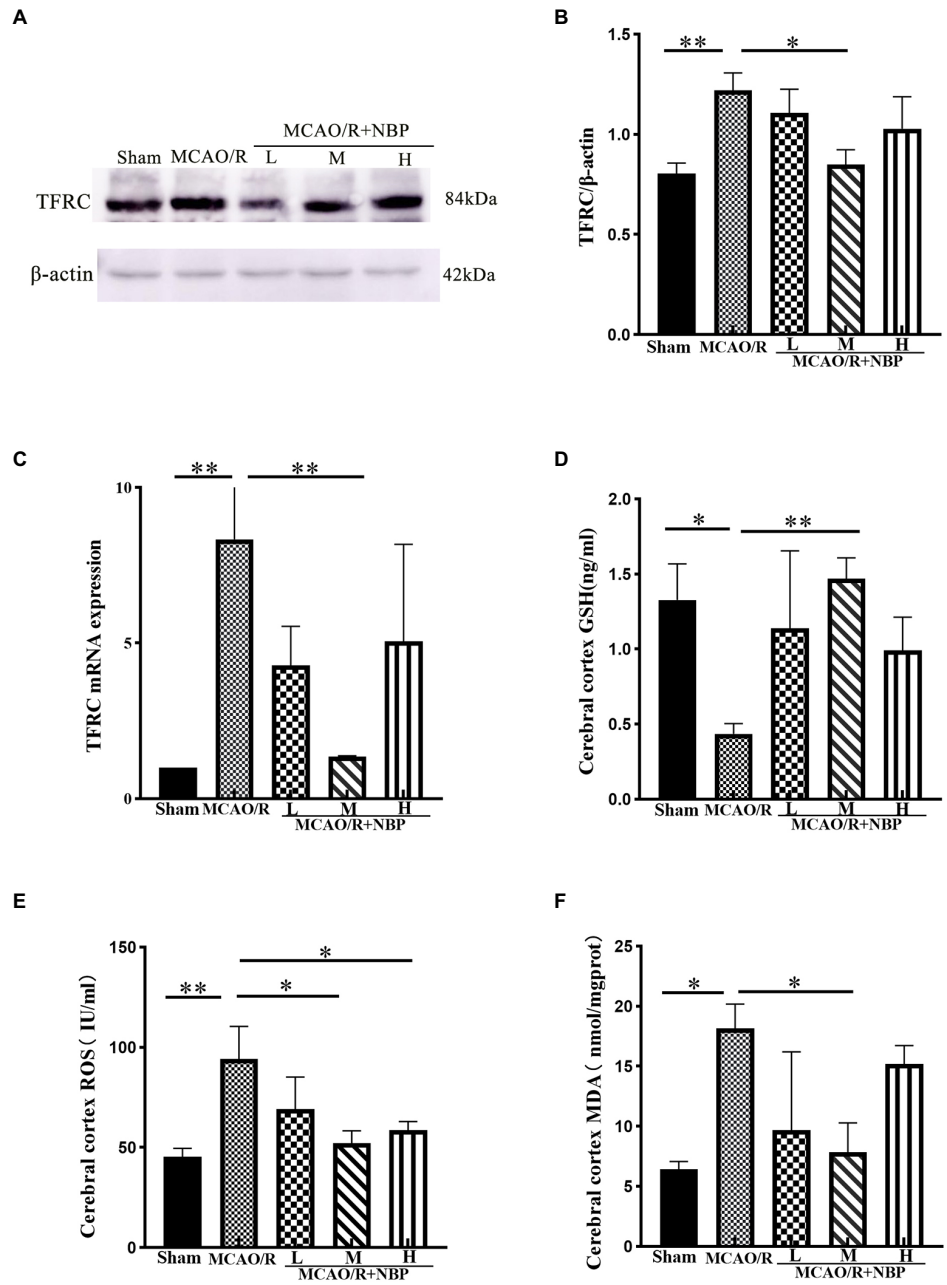
The relative levels of ferroptosis-related proteins were examined by Western blotting and ELISA. **(A)** Representative western blot image of TFRC. **(B)** Quantification of TFRC. **(C)** PCR for TFRC. **(D–F)** The result of GSH, ROS, and MDA by ELISA kit. ELISA, enzyme-linked immunoassay kit; TFRC, transferrin receptor; GSH, glutathione; ROS, Superoxide dismutase; MDA, malondialdehyde; PCR, real-time polymerase chain reaction. *p < 0.05, **p < 0.01, ***p < 0.001. The values represent the mean ± SD, *n* = 3.

TFRC is a critical protein for intracellular iron ion transfer, and our experimental results showed a significant increase in the expression of TFRC (*p* < 0.01, 1.220 ± 0.0870 vs. 0.8049 ± 0.0517; Figures 6A,B). This indicates that there are more free iron ions in cells after MCAO. After DL-NBP treatment, there were apparent changes (*p* < 0.05, 0.8489 ± 0.0748 vs. 1.220 ± 0.0870; Figure 6B). MCAO group TFRC (*p* < 0.01) levels rose compared to the Sham group in PCR. After applying DL-NBP, TFRC (*p* < 0.01, MCAO/R + NBP-M vs. MCAO, 1.355 ± 0.02738 vs. 8.338 ± 2.211; Figure 6C) was reduced. The ELISA method detected GSH, MDA, and ROS levels to indicate the ferroptosis-related correlates of cellular oxidative stress. Levels of ROS (*p* < 0.01, 94.24 ± 16.17 vs. 45.35 ± 4.174; Figure 6E) and MDA (*p* < 0.05, MCAO vs. Sham, 18.15 ± 2.029 vs. 6.424 ± 0.6414; Figure 6F) were significantly higher compared to that in the Sham group, while GSH levels were lower (*p* < 0.05, MCAO vs. Sham, 0.4365 ± 0.0683 vs. 1.327 ± 0.2412; Figure 6D). After treatment with DL-NBP, GSH was significantly upregulated (*p* < 0.01, MCAO/R + NBP-M vs. MCAO, 1.470 ± 0.1380 vs. 0.4365 ± 0.0683; Figure 6D), and ROS (*p* < 0.05, MCAO/R + NBP-M vs. MCAO, 52.08 ± 6.187 vs. 94.24 ± 16.17; Figure 6E) and MDA (*p* < 0.05, MCAO/R + NBP-M vs. MCAO, 7.853 ± 2.438 vs. 18.15 ± 2.029; Figure 6F) were downregulated.

### 3.6. Suppression of ferroptosis by DL-NBP is possibly mediated through SLC7A11/GSH/GPX4 signal pathway

**Figure 7:**
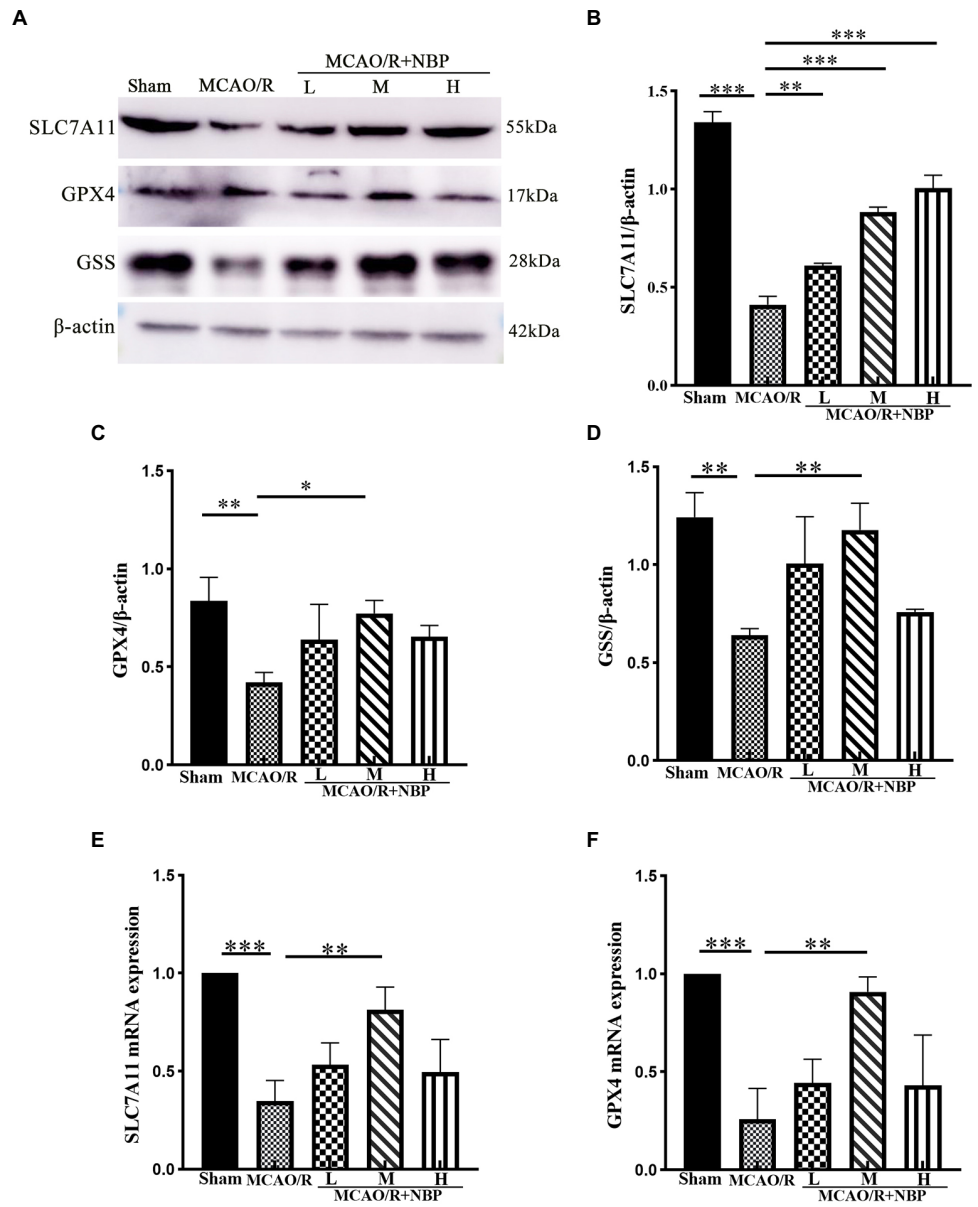
Western blot and PCR of ferroptosis-dependent SLC7A11/GSH/GPX4 signal pathway. **(A)** Representative western blot image of SLC7A11, GPX4, and GSS. **(B–D)** Quantification of SLC7A11, GPX4, and GSS. **(E,F)** PCR for SLC7A11, GPX4 levels within cerebral cortex tissues. PCR, Real-time polymerase chain reaction. *p < 0.05, **p < 0.01, ***p < 0.001. The values represent the mean ± SD, *n* = 3.

According to the western blotting results, SLC7A11 (*p* < 0.001, 0.4119 ± 0.0419 vs. 1.340 ± 0.0551), GPX4 (*p* < 0.01, 0.4215 ± 0.0498 vs. 0.8374 ± 0.1189), and GSS (*p* < 0.05, MCAO vs. Sham, 0.6400 ± 0.0347 vs. 1.244 ± 0.1248) were downregulated (Figures 7A–D). Compared to the MCAO/R group, SLC7A11 (*p* < 0.001, 0.8843 ± 0.0248 vs. 0.4119 ± 0.0419), GPX4 (*p* < 0.05, 0.7728 ± 0.0661 vs. 0.4215 ± 0.0498), and GSS (*p* < 0.01, 1.178 ± 0.1357 vs. 0.6400 ± 0.0347) in MCAO/R + NBP-M were upregulated after DL-NBP treatment. PCR shows the same conclusion. Compared to the Sham group, MCAO group SLC7A11 (*p* < 0.001) and GPX4 (*p* < 0.001) levels were lowered. After the application of DL-NBP, SLC7A11 (*p* < 0.01, MCAO/R + NBP-M vs. MCAO, 0.8129 ± 0.1165 vs. 0.3471 ± 0.1061) and GPX4 (*p* < 0.01, MCAO/R + NBP-M vs. MCAO, 0.9076 ± 0.07632 vs. 0.2593 ± 0.1559) were both increased (Figures 7E,F). The results showed that DL-NBP could probably mediate SLC7A11/GSH/GPX4 signal pathway to attenuate ferroptosis.

## 4. Discussion

Ischemic stroke is one of the most severe diseases worldwide and is associated with a poor prognosis and complications, including central nervous system infection and thrombosis of the deep veins (Pan et al., 2018; Yan W. et al., 2021). Dl-3-NBP, the main active ingredient in NBP, was initially isolated from celery seeds. As a new drug recommended for treating ischemic stroke in China, it has been shown to have antioxidant properties, reduce the inflammatory response, and promote angiogenesis (Zhao et al., 2014). It is a new therapy with multisite and multitarget regulation features, showing apparent neuroprotective effects. In this study, we built a model of brain ischemia–reperfusion to observe whether the SLC7A11/GSH/GPX4 and PDGFRβ/PIK/Akt signal pathways were involved in the process; most importantly, we evaluated whether these two pathways could be potential valid targets of DL-NBP treatment.

The results showed that the medium-dose DL-NBP alleviated MCAO-induced neurologic deficits, brain water content, and infarction area and improved nerve function and ischemic brain injury compared to the MCAO group. Previous research not only reached the same conclusion (Wang J. et al., 2021; Wei et al., 2021) but also proved that BBB destruction could increase tissue swelling and lead to brain damage (Song et al., 2022). These three studies confirm the successful establishment of a model of hypoxic–ischemic brain injury but also serve as a reminder of the damage to the BBB in this injury, which has also been mentioned in other articles (Zhu et al., 2021). In the meantime, these outcomes also remind us that an appropriate dose of DL-NBP is an effective treatment method for ischemic stroke. We also observed through HE staining that nuclear vacuole formation, atomic condensation, contraction, and nuclear lysis were reduced with DL-NBP treatment. The neuronal cells after Nissl staining had a square shape. TUNEL staining showed reduced apoptotic cells (Pi et al., 2021). Ferroptosis is another type of cell death (Gao et al., 2016). Whether DL-NBP could affect ferroptosis in MCAO/R is another question for future research. The conditions of DL-NBP-administered groups improved compared to the MCAO group in the study. Therefore, the targets of BBB and ferroptosis that DL-NBP works on were the main focus of this article.

The PI3K/Akt pathway is an essential intracellular signaling pathway involved in processes such as cell resting, proliferation, and cancer (Xie et al., 2019; Feng H. et al., 2020). PDGF-β is expressed in cerebral mesenchymal cells. PDGFR-β was found to function in endothelial progenitor cells overexpressed by the PI3K/Akt signaling pathway to exert neuroprotective functions in adult mice (Zheng et al., 2019). PDGFRβ activates the PI3K-AKT pathway and has roles on the cell membrane, including promoting actin recombination, directing cell proliferation, stimulating cell growth, improving angiogenesis, and inhibiting apoptosis (Fan et al., 2014). It is also an essential protective pathway for the BBB after MCAO/R-induced brain damage (Shen et al., 2019). The binding of PDGF to PDGFRβ by activating the PI3K-Akt signaling pathway in rat models can enhance cell proliferation and migration, promoting early vascular repair and angiogenesis after cerebral ischemia–reperfusion injury (Zheng et al., 2019). Our experiment observed the decreasing expression of p-PI3K and p-Akt and the increased expression of PDGFRβ after MCAO. We inferred that, along with the weak signal of the PDGFRβ/PIK/Akt pathway, it was unable to promote the binding of PDGF to PDGFRβ, resulting in the upregulation of PDGFRβ expression. This is consistent with previous research (Zhang et al., 2007). After treatment with DL-NBP, PDGFRβ was downregulated and p-PI3K, as well as p-Akt, experienced elevated expression. In addition, DL-NBP preserved the expression of tight junction proteins ZO-1 and occludin in our experiment. The same results were seen in the immunofluorescence double-label CD31 with ZO-1 or occludin. Microvascular endothelial cells are the critical component of BBB, and platelet endothelial cell adhesion molecule-1 (CD31) is expressed in it. Previous research has proved that ZO-1 and occludin are essential tight junction proteins in the BBB structure, maintaining its stabilization, and their absence could destroy the BBB directly (Song et al., 2022). Co-immunostaining ZO-1 (or occludin) with CD31 could detect the integrity of the tight junction of the BBB better. The density of luminous dots was observed from immunofluorescence staining, and we showed coincident results with western blotting after quantification. This conclusion is similar to the BBB permeability shown by EB staining. These outcomes indicate that DL-NBP likely promotes the recovery of BBB *via* PDGFRβ/PI3K/Akt. We conclude from previous research (Shen et al., 2019) and our experiment that DL-NBP could presumably mediate the PDGFRβ/PIK/Akt signal pathway and alleviate the damage of the BBB caused by ischemia–reperfusion injury.

This study found the downregulation of SLC7A11, GPX4, GSH, and GSS after MCAO and the upregulation of TFRC by western blotting and an ELISA kit. However, the medium DL-NBP treatment group had a better outcome than the MCAO group. Transferrin receptor (TFRC) is a receptor on the cell membrane that transfers free iron ions into cells, which is regarded as a sign of ferroptosis (Feng H. et al., 2020). Owing to neuronal dysfunction, TFRC levels increased as it lost its ability to bond with free iron ions. The ferroptosis-related proteins were also changed, such as the downregulation of the SLC7A11/GSH/GPX4 signal pathway proteins; DL-NBP appeared to have helped in this. Protein expression significantly changed, which was consistent with the histopathological findings. This indicates that SLC7A11/GSH/GPX4 was probably one of the targets for DL-NBP to cure ischemic stroke.

Substrate-specific subunit SLC7A11 and auxiliary regulatory subunit SLC3A2 form the main pump of the cysteine glutamate antiporter (system xc-), which ingests cystine into cells to converts it into cysteine for GSH synthesis and pumps out high concentration glutamate intracellularly (Lin et al., 2022). SLC7A11-imported cystine is the raw material for GSH biosynthesis, which promotes subsequent GPX4-mediated lipid peroxidase detoxification to inhibit ferroptosis (Chen et al., 2021b). SLC7A11 is essential in transferring antioxidant raw material, and GPX4 is a critical cellular enzyme involved in the regulation of ferritin growth, which can directly reduce lipid peroxide to non-toxic alcohols in the membrane to prevent ferroptosis in the process of lipid oxidation (Hambright et al., 2017; Koppula et al., 2021). Research has shown that the deletion of SLC7A11 or GPX4 in mice could cause ferroptosis-like damage, lead to cognitive impairment and neurodegeneration, and even show early embryonic lethality (Koppula et al., 2021).

GSH is the crucial link in the SLC7A11/GSH/GPX4 signal pathway. Under normal conditions, systematic xc-transporters on neuronal cell membranes can exchange the extracellular GSH raw material cystine with intracellular glutamate in cells (Mandal et al., 2010). Under the catalysis of GPX4 and GSS (GSH synthase), two molecules of GSH are oxidized to produce one molecule of GSH disulfide (GSSG) (Chen et al., 2021a). This reaction provided electrons to react with ROS and reactive nitrogen species such as hydrogen peroxide (H_2_O_2_) or organic peroxides (ROOH) to reduce into water or non-toxic alcohols (Lin et al., 2022). The process could maintain the intracellular antioxidant system and redox balance. GSS-defective mice cannot survive the embryonic stage, suggesting that GSH is essential for embryogenesis (Aoyama, 2021). In addition, GSH depletion in the brain is a common finding in patients with neurodegenerative diseases such as Alzheimer’s and Parkinson’s disease (Aoyama, 2021; Shi et al., 2022).

The SLC7A11/GSH/GPX4 signal pathway is the fundamental element of intracellular antioxidant function, which regulates the production and balance of antioxidants in the intracellular space (Cao and Dixon, 2016). Our research also confirmed these findings to reinstate the importance of the SLC7A11/GSH/GPX4 antioxidant axis in ferroptosis, which aligns with previous studies (Bu et al., 2021). The appropriate dose of DL-NBP has the probable function of targeting the SLC7A11/GSH/GPX4 signal pathway to treat the disease. This provides a reference for future clinical treatment of ischemic stroke. Whether DL-NBP affects the ferroptosis signal pathway could also be shown by MDA content (Zhao et al., 2014) and ROS. MDA is an aldehyde byproduct of lipid peroxidation, which can help measure the degree of lipid peroxidation and is one of the markers of ferroptosis (Wang Y. et al., 2021). There are several sources of ROS, including the oxygen in restored blood flow during the reperfusion phase, where highly active alkoxy radicals (L-O•) produced by L-OOH are oxidized by free iron ions (She et al., 2020), and a decrease in antioxidants such as GSH (Zhou et al., 2020) is observed. We could infer from the results of the MDA and ROS data in our experiment that DL-NBP could reduce the content of both in the MCAO/R brain tissue. This is indirect evidence that DL-NBP tends to alleviate the ferroptosis pathway.

Through our experiments, the brain tissue GSH in the MCAO group decreased significantly, while ROS and MDA increased compared with the Sham group. After treatment with medium-dose DL-NBP, the GSH upregulation was prominent, while ROS and MDA declined significantly. The oxidative stress state of neuronal cells was reduced following treatment with DL-NBP. Previous studies have also shown the antioxidative stress effects of DL-NBP in rat renal ischemic reperfusion (Dong et al., 2021; Wang B. N. et al., 2021), similar to our experimental findings.

Ferroptosis is evoked by iron-dependent lipid peroxidation, which has the characteristics of intracellular iron overload, GSH deficiency, and GPX4 dysfunction that can lead to redox disorders and partial tissue dysfunction (Cao and Dixon, 2016). After a stroke, increased free iron ions and rich polyunsaturated fatty acid (PUFA) in neuronal membranes provide the conditions for the occurrence of ferroptosis in brain tissue (Yan et al., 2020). The regular operation of the oxidative-resistant SLC7A11/GSH/GPX4 signaling pathway is interrupted in this situation. The increased free iron ions and rich PUFA contribute to ferroptosis (She et al., 2020). DL-NBP could presumably alleviate ferroptosis by affecting SLC7A11/GSH/GPX4 pathway. This is consistent with the comparison of results between the MCAO/R and MCAO/R + NBP-M groups in this experiment.

In summary, a series of oxidative stresses occur after cerebral ischemia and reperfusion. These stresses reduce the cellular antioxidant GSH and lead to the accumulation of ROS and MDA, leading to dysfunction of the SLC7A11/GSH/GPX4 pathway of neuronal cells, which ultimately mediates cell death. At the same time, due to the imbalance of the PDGFRβ/PI3/Akt pathway, the recovery of the endothelial structures of the blood–brain barrier is hindered. DL-NBP has been used in clinical settings to address psychiatric and behavioral functions following acute ischemic stroke in China (Fan et al., 2021). Our research has provided further support for using DL-NBP therapy in this setting (Yang et al., 2021).

DL-NBP could be used as a co-treatment method along with thrombolysis or mechanical thrombectomy treatment. Early initiation of neuron cytoprotecting, especially in the prehospital period (e.g., ambulance), would target the ischemic penumbra to slow down its evolution into the infarct zone (Savitz et al., 2019; Lyden et al., 2021). Meanwhile, it could also be considered to be used in-hospital pre-thrombectomy and for post-thrombectomy cytoprotection (Savitz et al., 2019).

This study had a few limitations. Our experiments show that DL-NBP can influence the SLC7A11/GSH/GPX4 and PDGFRβ/PI3/Akt pathways to alleviate ferroptosis and damage to the BBB, but a specific cell type has not yet been identified. We also did not use inhibitors to provide comparisons in the experiment. Although we had considered the feasibility of experimental surgery and bias based on sex, geriatric disease, and gender incidence are still our limitations in this experiment. These limitations should be addressed in future studies.

## 5. Conclusion

In general, our findings suggest that DL-NBP is likely to reduce ferroptosis, inflammatory response, apoptosis, and BBB permeability of brain tissue cells to protect brain tissue from ischemia–reperfusion damage in a rat model of ischemic stroke. We found that DL-NBP could presumably mediate the SLC7A11-GSH-GPX4 and PDGFRβ/PI3/Akt pathways. The expressions of the essential proteins and genes to produce this neuroprotective effect suggests that this signaling pathway may be the mechanism behind this protective effect. The results of this study provide a reference for the clinical neuroprotective effect of DL-NBP.

## Data availability statement

The original contributions presented in the study are included in the article/Supplementary material, further inquiries can be directed to the corresponding authors.

## Ethics statement

The animal study protocol was reviewed and approved by the experimental animal Ethics Committee of Weifang Medical University 77 (2018-037).

## Author contributions

SX, XuL, and YW conceptualized and designed the experiments. SX drafted the article and performed most of the experiments. YL, XuL, XiL, EL, and XZ conducted experiments. YS and YW made the approval of the final manuscript. All authors contributed to the article and approved the submitted version.

## Funding

This work was supported by the National Natural Science Foundation of China (No. 81870943) and Yuan Du Scholars.

## Conflict of interest

The authors declare that the research was conducted in the absence of any commercial or financial relationships that could be construed as a potential conflict of interest.

## Publisher’s note

All claims expressed in this article are solely those of the authors and do not necessarily represent those of their affiliated organizations, or those of the publisher, the editors and the reviewers. Any product that may be evaluated in this article, or claim that may be made by its manufacturer, is not guaranteed or endorsed by the publisher.
