# Supplementary material for: Neuroprotective effect of Dl-3-n-butylphthalide against ischemia–reperfusion injury is mediated by ferroptosis regulation via the SLC7A11/GSH/GPX4 pathway and the attenuation of blood–brain barrier disruption
Source: Front Aging Neurosci. 2023 Feb 23;15:1028178. doi: 10.3389/fnagi.2023.1028178 (PMC9995665; doi:10.3389/fnagi.2023.1028178)
Supplement: Supplementary file 1 [file Data_Sheet_2.PDF]

Neuroprotective effect of Dl-3-n-butylphthalide against ischemia-reperfusion injury is mediated by ferroptosis regulation via the SLC7A11/GSH/GPX4 pathway and the attenuation of blood-brain barrier disruption

Shuangli Xu[1], Xuewei Li[2], Yutian Li[3], Xiangling Li[4], E Lv[5], Xiaojun Zhang[2], Youkui Shi[1*], Yanqiang Wang[2*]

[1]Emergency Department, the Affiliated Hospital of Weifang Medical University, Weifang, Shandong, China
[2]Department Ⅱ of Neurology, the Affiliated Hospital of Wei fang Medical University, Weifang, Shandong, China
[3]School of Pharmacy, Weifang Medical University, Weifang, Shandong, China.
[4]Department of Internal Medicine, the Affiliated Hospital of Weifang Medical University, Weifang, Shandong, China
[5]Department of Histology and Embryology, Weifang Medical University, Weifang, Shandong, China.

***Correspondence:**
Youkui Shi
E-mail: syk2101603@163.com
Yanqiang Wang
E-mail: wffywyq2006@126.com or wangqiangdoctor@126.com

Running title: The neuroprotective effect of Dl-3-n-butylphthalide

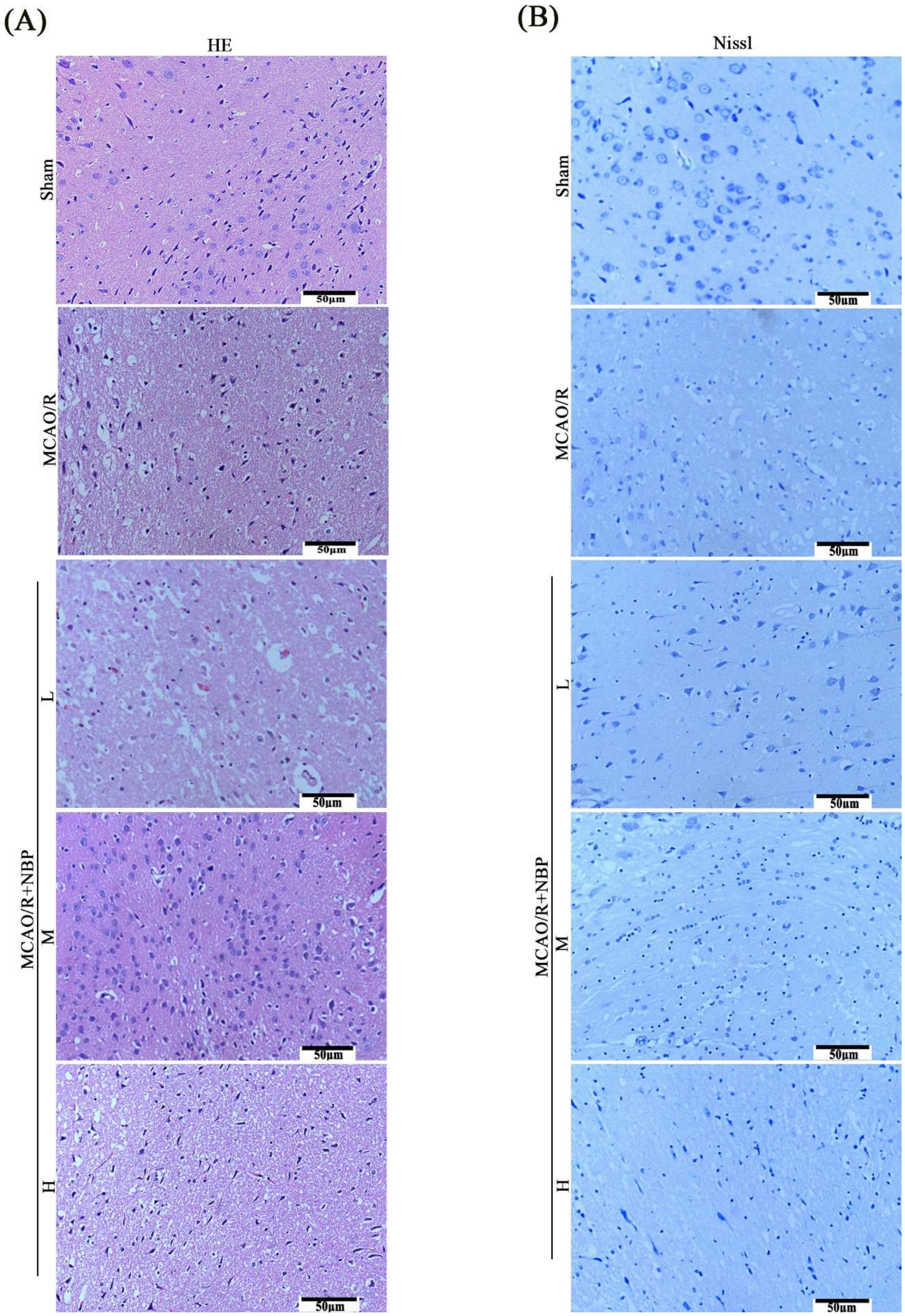

Supplementary Figure 1. The histopathological and structural changes by HE, Nissl staining. (A) HE staining, x200. (B) Nissl staining, x200.

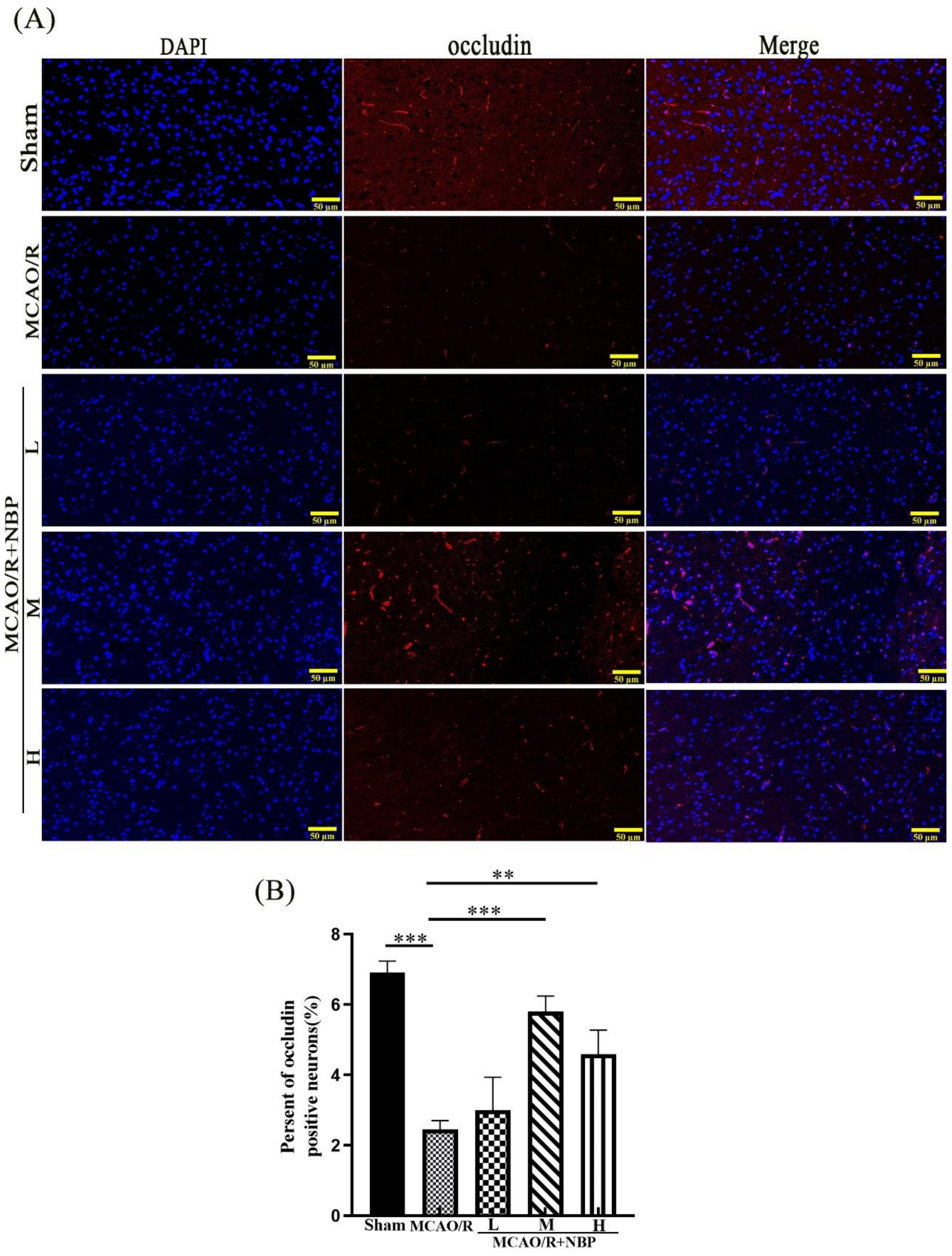

Supplementary Figure 2. The occludin tight junction protein of BBB. (A) The expression of occludin. (B) The qualification of occludin. BBB, the blood-brain barrier. *, $p< 0.05$; **, $p < 0.01$; ***, $p<0.001$. The values represent the mean ± SD, n = 3.

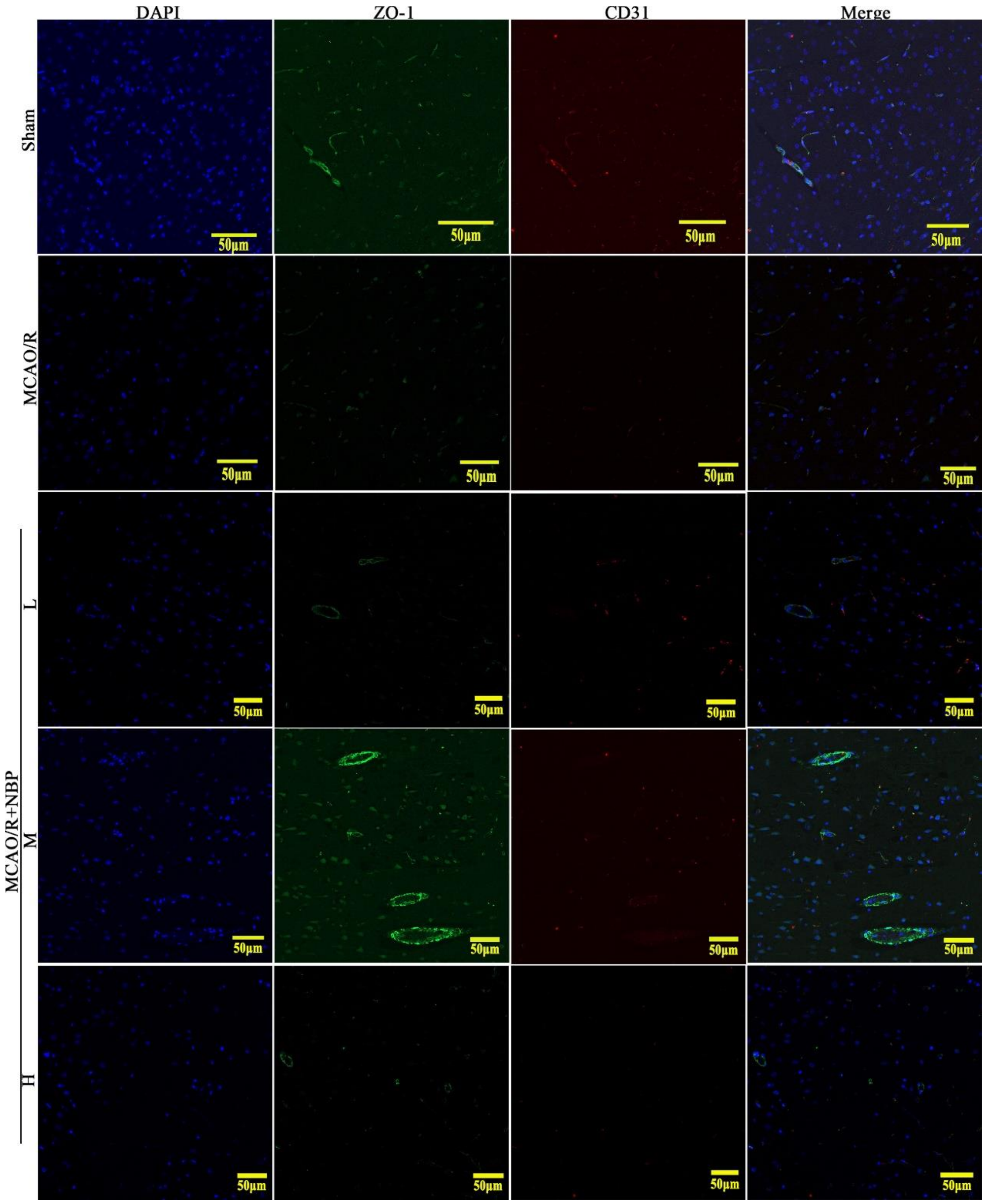

Supplementary Figure 3. Co-immunostaining ZO-1 with CD31 could detect the integrity of the tight junction of the BBB better. The density of luminous dots is observed from immunofluorescence staining. The immunofluorescence double-label CD31 with ZO-1.

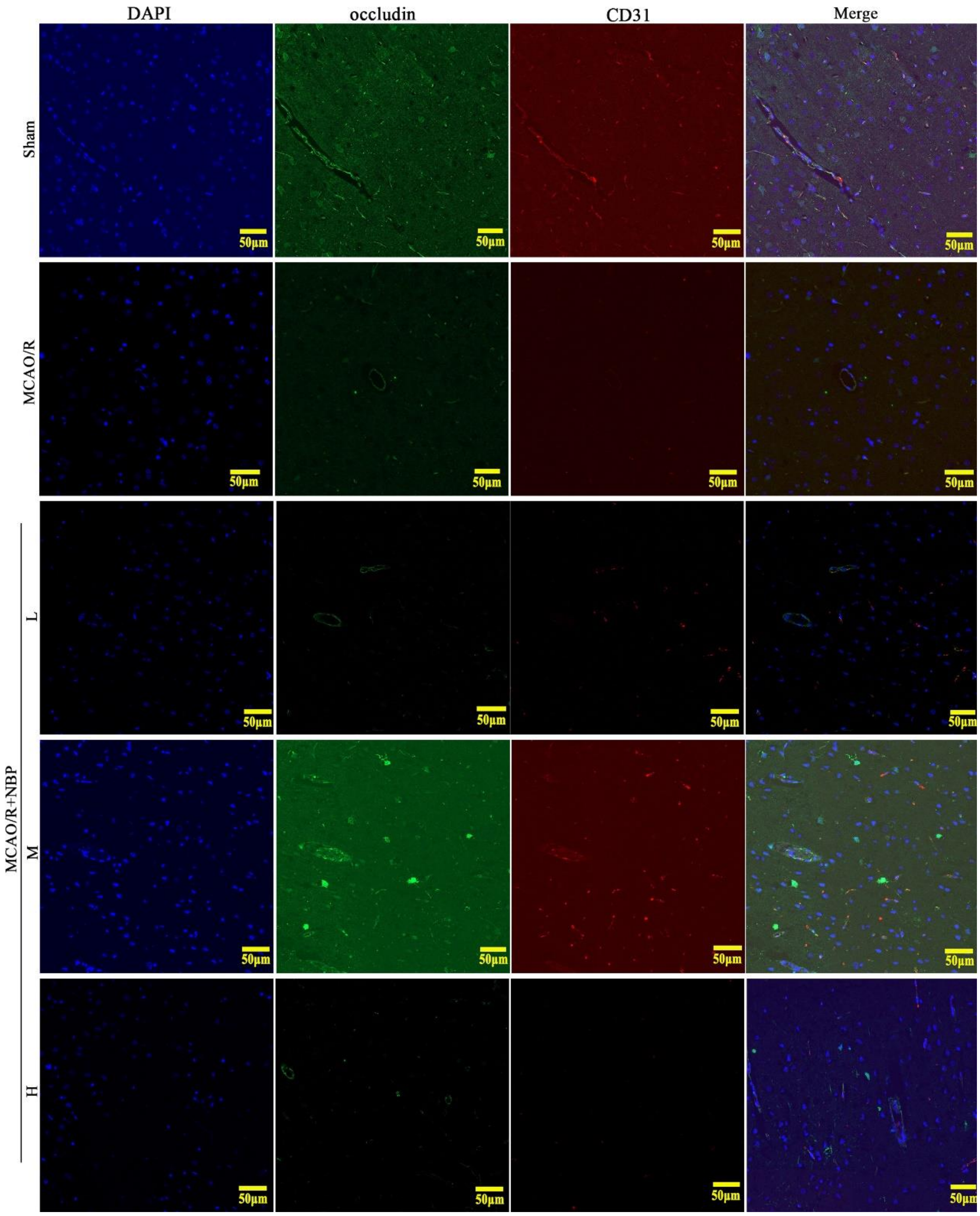

Supplementary Figure 4. Co-immunostaining occludin with CD31 could detect the integrity of the tight junction of the BBB better. The density of luminous dots is observed from immunofluorescence staining. The immunofluorescence double-label CD31 with occludin.

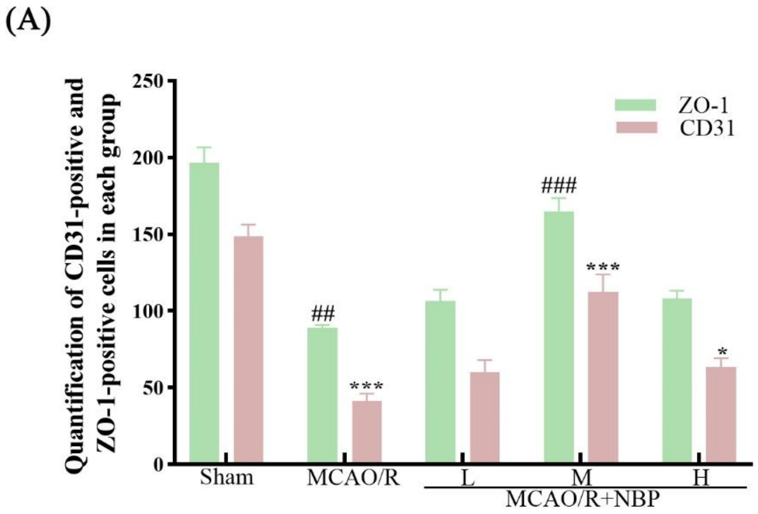

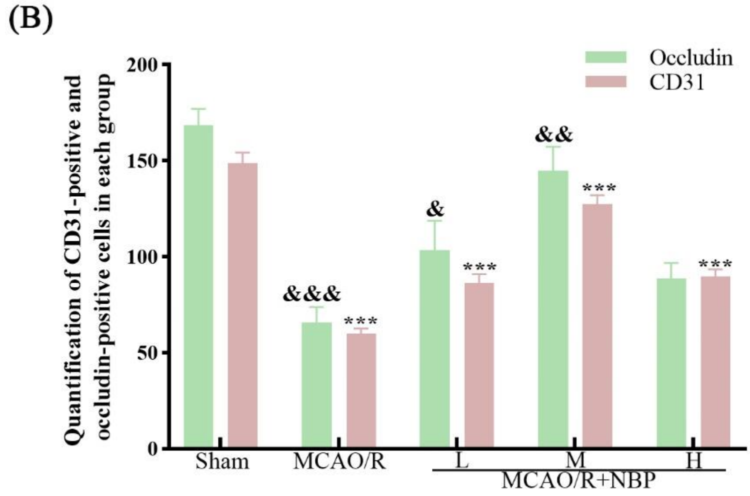

Supplementary Figure 5. (A) The qualification of CD31-positive and ZO-1-positive cells in each group. (B) The qualification of CD31-positive and occludin-positive cells in each group. ##, p<0.001(Sham vs MCAO/R); ###, p<0.001(MCAO/R vs MCAO/R+NBP-M); &, p<0.05(MCAO/R vs MCAO/R+NBP-L); &&, p<0.001(MCAO/R vs MCAO/R+NBP-M); &&&, p<0.001(Sham vs MCAO/R); p *, p<0.05; ***, p<0.001. Data are presented as the mean ± SD, n = 3.

https://www.jianguoyun.com/p/DbXtNOYQn_jrChiQ6NAEIAA

The original data has been shown in JIANGUOYUN.
